# POFUT1 Serves as an Independent Prognostic Factor and Therapeutic Target by Activating the PI3K/AKT Pathway in Glioma

**DOI:** 10.1002/brb3.71701

**Published:** 2026-08-31

**Authors:** Kai Yang, Chunlei Ju, Yifan Wang, Aijie Guo, Xiaochen Yang, Yang Mi, Yifei Su, Chunhong Wang, Hongming Ji

**Affiliations:** ^1^ Department of Neurosurgery Shanxi Provincial People's Hospital Shanxi Medical University Taiyuan China; ^2^ The Neurosurgery Department, Xi'an Xidian Hospital Xi'an China

**Keywords:** glioma, PI3K, POFUT1, prognosis

## Abstract

**Objective:**

Protein O‐fucosyltransferase 1 (POFUT1) has been implicated in several malignancies, but its functional and prognostic significance in glioma remains insufficiently defined. This study evaluated whether POFUT1 expression is associated with glioma progression, patient outcome, and PI3K/AKT pathway activity.

**Methods:**

Public glioma transcriptome datasets from The Cancer Genome Atlas (TCGA) and Chinese Glioma Genome Atlas (CGGA) were analyzed and compared with clinical samples collected from 123 glioma patients. POFUT1 protein levels in clinical specimens were determined by immunohistochemical staining, and its association with patient outcome was analyzed using survival curves. In vitro, glioma cell growth, motility, and invasiveness were examined using MTT and Transwell assays. The effect of POFUT1 on tumor formation was further tested in a subcutaneous xenograft model. RNA sequencing, KEGG pathway enrichment, and pharmacological inhibition were then used to explore the mechanism linking POFUT1 to PI3K/AKT signaling.

**Results:**

POFUT1 expression was higher in glioma than in normal brain tissue and increased with tumor grade. Patients with high POFUT1 levels had shorter overall survival, and multivariate Cox analyses supported POFUT1 as an independent prognostic indicator. Incorporating POFUT1 into a nomogram improved prediction of 1‐, 3‐, and 5‐year survival. Functionally, POFUT1 knockdown reduced glioma cell growth, motility, invasion, and xenograft expansion, whereas POFUT1 overexpression produced the opposite phenotype. Transcriptomic and protein analyses indicated that POFUT1 enhanced PI3K/AKT signaling. The PI3K inhibitor LY294002 weakened the tumor‐promoting effects caused by POFUT1 overexpression.

**Conclusion:**

POFUT1 as a key driver of glioma malignancy, predominantly through activating the PI3K‐AKT signaling pathway. These findings highlight POFUT1 as a promising novel therapeutic target for aggressive glioma.

## Graphical abstract

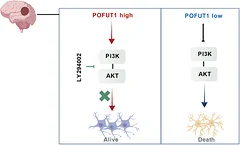

**Abbreviations**
POFUT1: protein O‐fucosyltransferase 1
CGGA: Chinese Glioma Genome Atlas
IDH: isocitratedehydrogenase
MGMT: O6‐methylguanine‐DNA methyltransferase
ROC: receiver operating characteristic
IHC: immunohistochemistry

## 1 Introduction

Gliomas are the most common malignant tumors arising in the central nervous system and are among the most difficult intracranial neoplasms to treat (Omuro 2013). Their diffuse infiltration into surrounding brain tissue, marked biological heterogeneity, and frequent recurrence contribute to unsatisfactory clinical outcomes (Wu et al. 2025). At present, the main therapeutic strategy consists of maximal safe resection combined with postoperative radiotherapy and temozolomide‐based chemotherapy (Xu et al. 2020). Although this regimen has become the standard of care, its efficacy is still limited, particularly in highly aggressive glioma subtypes such as glioblastoma. In recent years, molecular features, including isocitrate dehydrogenase (IDH) mutation, 1p/19q co‐deletion, and O6‐methylguanine‐DNA methyltransferase (MGMT) promoter methylation, have been incorporated into glioma diagnosis and classification (Śledzińska et al. 2021). These advances have improved prognostic assessment and facilitated more precise disease stratification. Meanwhile, emerging therapeutic strategies have been actively explored to overcome treatment resistance (Yasinjan et al. 2023; Wu et al. 2025). However, the complex molecular landscape of glioma and its immunosuppressive tumor microenvironment continue to restrict durable therapeutic responses, underscoring the need to identify novel regulatory mechanisms involved in glioma progression.

As a pivotal glycosyltransferase, POFUT1 modifies Notch receptors by adding O‐linked fucose to their epidermal growth factor‐like repeat domains (Wu et al. 2025). This modification is required for appropriate receptor maturation, membrane trafficking, ligand recognition, and downstream Notch signal activation (Li et al. 2021). As a cancer‐associated glycosylation enzyme, POFUT1 has been reported to participate in malignant progression by regulating key signaling pathways, particularly Notch signaling (Pennarubia et al. 2022). Aberrant POFUT1 expression has been implicated in multiple tumor types, including breast, colorectal, and lung cancers (Mazour et al. 2025). In these malignancies, POFUT1 may enhance cell proliferation, stem‐like properties, metastatic potential, and therapeutic resistance (Dong et al. 2023; Li et al. 2024). A previous study proposed that POFUT1 can promote glioblastoma malignancy through Notch pathway activation. Nevertheless, it is still unclear whether POFUT1 has independent prognostic value in glioma and whether additional signaling pathways participate in POFUT1‐mediated tumor progression.

The PI3K‐AKT pathway is a central signaling cascade that governs cell growth, survival, metabolism, and stress adaptation (Noorolyai et al. 2019). Constitutive activation of this pathway is common in human cancers and contributes to aggressive tumor behavior and treatment resistance. In glioblastoma, abnormal PI3K/AKT activity is frequently associated with poor outcome (Noorolyai et al. 2019; Tang et al. 2023). Thus, targeting this pathway has emerged as an important strategy for therapeutic exploration in glioma.

Despite its established role in glioma, the upstream mechanisms regulating PI3K‐AKT activation remain incompletely understood. Whether POFUT1 contributes to glioma progression by modulating this pathway is still unclear. Elucidating the POFUT1/PI3K‐AKT axis may provide new insight into glioma malignancy and reveal potential therapeutic targets.

## 2 Materials and Methods

### 2.1 Database

RNA‑sequencing data were obtained from two publicly available databases. The CGGA mRNAseq_693 and mRNAseq_325 cohorts, containing 693 and 325 cases, respectively, served as validation datasets. TCGA glioma data, including glioblastoma (GBM) and lower‐grade glioma (LGG) samples, were used for complementary analysis.

### 2.2 Patient Samples

This study analyzed 115 surgically resected glioma tissues and 8 normal brain tissues collected at Shanxi Provincial People's Hospital. All glioma samples were obtained from patients who underwent tumor resection without receiving any preoperative antitumor treatment, including radiotherapy, chemotherapy, or immunotherapy. Detailed clinicopathological characteristics were recorded and are provided in Table S1.

### 2.3 Cell Culture

Human GBM cell lines U251‐MG (U251) and U87‐MG (U87) were obtained from Hunan Fenghui Biotechnology Co., Ltd. Cells were cultured in complete DMEM medium supplemented with fetal bovine serum and penicillin–streptomycin under conventional laboratory conditions.

### 2.4 Knockdown and Overexpress POFUT1 in Glioma Cell Lines

For POFUT1 silencing, glioma cells were transfected with POFUT1‐targeting small interfering RNAs (siRNA) or corresponding control using Lipofectamine 2000 (Thermo, USA). The siRNA sequences are listed in Table S2. For POFUT1 overexpression, cells were infected with lentiviral vectors carrying the full‐length POFUT1 coding sequence, and empty vectors were used as controls.

### 2.5 Immunohistochemistry (IHC)

Immunohistochemistry was performed on paraffin‐embedded tissue sections to determine POFUT1 protein expression. The staining procedure was carried out following routine laboratory protocols. After incubation with the POFUT1 primary antibody and the corresponding secondary antibody, immunoreactivity was visualized by chromogenic staining and observed microscopically. The staining results were evaluated semi‐quantitatively by considering both signal intensity and the percentage of positive tumor cells, and POFUT1 expression was represented by H‐scores.

### 2.6 Western Blot

Total cellular protein was isolated using RIPA buffer containing protease inhibitors, and protein concentrations were measured with a BCA assay kit (Boster Biological Technology, China). Equal amounts of protein (40 µg) were resolved by SDS‐PAGE and transferred to PVDF membranes (Millipore, USA). The membranes were incubated overnight at 4°C with primary antibodies against GAPDH (Proteintech, 1:2000), POFUT1 (Proteintech, 1:3000), ITGA5 (Abmart, 1:2000), THBS1 (Abmart, 1:1000), AKT (Abmart, 1:1000), p‐AKT (Abmart, 1:5000), PI3K (HUABIO, 1:1000), and p‐PI3K (Abmart, 1:1000). After incubation with anti‐rabbit or anti‐mouse secondary antibodies (Invitrogen, USA, 1:10,000) for 1.5 h, protein bands were visualized using an Odyssey CLx Imaging System (LI‐COR, USA).

### 2.7 Real‐Time Quantitative‐PCR (RT‐qPCR)

RT‐qPCR was used to measure the mRNA expression level of POFUT1 in glioma cells. Briefly, cellular RNA was prepared using TRIzol reagent, and qualified RNA samples were converted into cDNA with a reverse transcription kit. Quantitative PCR amplification was performed using SYBR Green chemistry. GAPDH served as the endogenous control, and relative POFUT1 expression was analyzed using the comparative 2^−ΔΔCt^ method. Detailed primer sequences are listed in Table S2.

### 2.8 MTT Assay

The effect of POFUT1 on glioma cell growth was evaluated using the MTT assay. Briefly, cells from different treatment groups were seeded at 5 × 10^3^ cells per well and cultured for the indicated time periods. MTT reagent was then added to assess cellular metabolic activity. After incubation, the generated formazan crystals were dissolved with DMSO. Absorbance was measured at 490 nm using a microplate reader, and the obtained optical density values were used to compare cell proliferation among groups.

### 2.9 Transwell Assays

Cell migration and invasion were analyzed using Transwell chambers. Briefly, 4 × 10^4^ cells in serum‐free medium were added to the upper chambers. For migration assays, uncoated inserts were used, while Matrigel‐precoated inserts (BD Biosciences, USA) were applied for invasion assays. The lower chambers were filled with complete medium containing 10% FBS as a chemoattractant. After 24 h of incubation, cells remaining on the upper surface of the membrane were removed, whereas cells that had passed through the membrane were fixed with 4% paraformaldehyde and stained with 0.1% crystal violet. The migrated or invaded cells were then photographed and quantified in five randomly selected microscopic fields.

### 2.10 Animal Xenograft Model

Four‐week‐old male BALB/c nude mice were used for in vivo tumorigenicity assays and were randomly divided into different experimental groups, with five animals in each group. U251 cells stably expressing POFUT1 or the corresponding control vector were collected and suspended in PBS before implantation. For tumor establishment, 1 × 10^7^ cells were injected subcutaneously into each mouse. LY294002 intervention began 7 days after cell inoculation, and the inhibitor was administered intraperitoneally at 25 mg/kg once every 3 days. Tumor dimensions were recorded every 3 days, and volumes were determined using the formula V = length × width× width /2. At the end of the study, tumors were removed, photographed, and weighed.

### 2.11 RNA Sequencing

Total RNA was extracted from cells with TRIzol reagent and subsequently delivered to BGI Genomics Co., Ltd. for transcriptome sequencing. RNA quality, concentration, purity, and integrity were first examined to ensure suitability for sequencing. Qualified RNA samples were then used to prepare sequencing libraries, which were analyzed by paired‐end sequencing on an Illumina platform. Clean sequencing reads were obtained after filtering out adaptor‐containing and low‐quality reads and were subsequently aligned against the human reference genome. Expression quantification and differential expression analysis were then performed based on the mapping results. Genes meeting the criteria of |log2 fold change| > 1 and FDR < 0.05 were regarded as significantly differentially expressed. The generated RNA‐seq dataset is publicly available in GEO with the accession ID GSE316535.

### 2.12 Statistical Analysis

Data from quantitative experiments were summarized as the mean ± standard deviation. For comparisons involving two groups, Student's t‐test was applied, while one‐way ANOVA was used when three or more groups were analyzed. The association between POFUT1 expression and clinical features was examined using Pearson's chi‐square test. Patient survival was evaluated using Kaplan–Meier curves, and differences between survival curves were assessed with the log‐rank test. Cox proportional hazards regression was performed to explore variables associated with prognosis. ROC curve analysis was used to estimate the predictive performance of the prognostic model. Statistical significance was defined as a two‐sided *p* value below 0.05.

## 3 Results

### 3.1 POFUT1 Is Overexpressed in Glioma and Correlates With Tumor Grade

**FIGURE 1:**
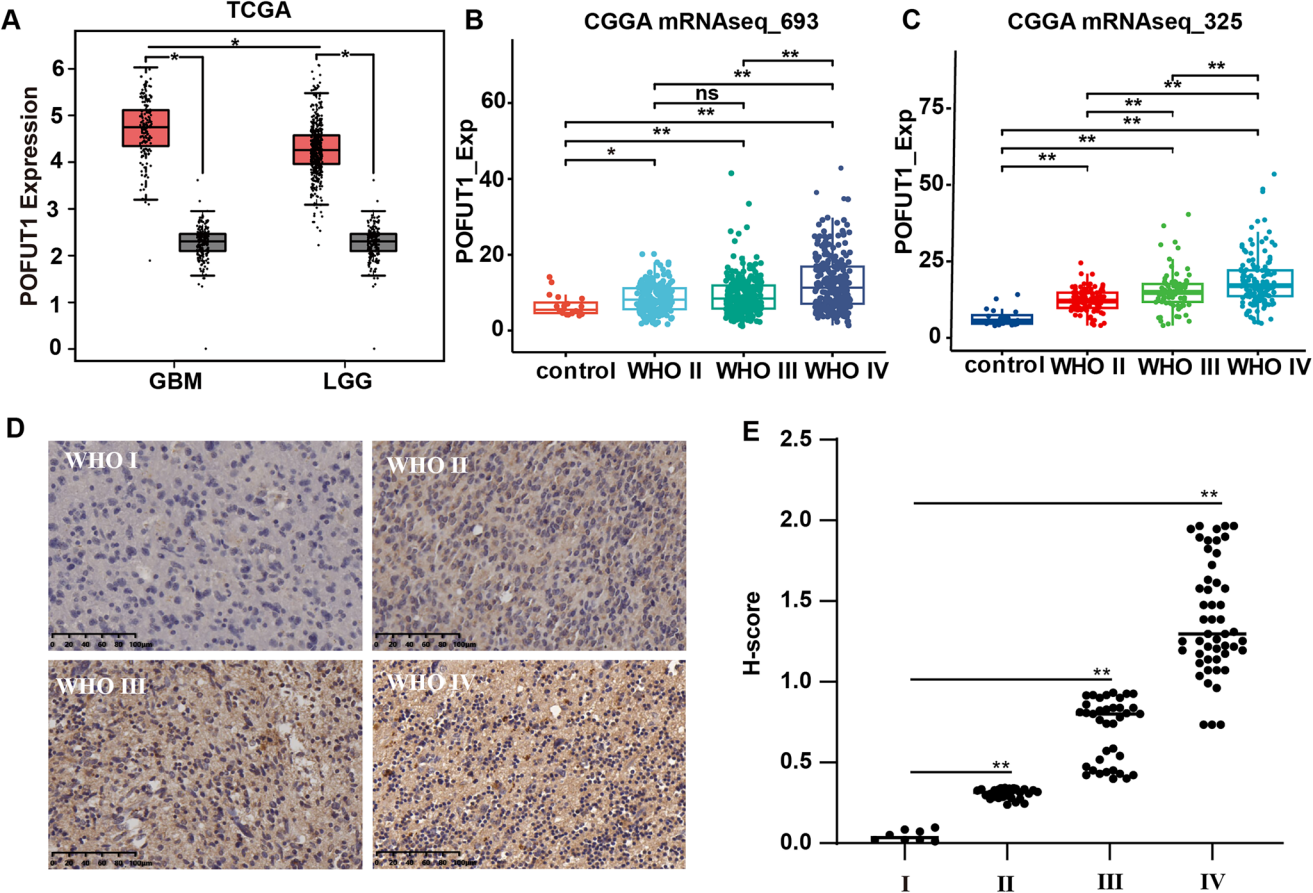
The expression of POFUT1 in gliomas. (A) The expression of POFUT1 in glioblastoma multiforme (GBM) and low‐grade glioma (LGG). The data from TCGA database. The expression of tumor is highlighted in red, the expression of normal is highlighted in black. *p < 0.05. (B) The expression of POFUT1 in GBM of different grades. The data from CGGA database mRNAseq_693. ns, *p* > 0.05, *p < 0.05, **p < 0.01. (C) The expression of POFUT1 in GBM of different grades. The data from CGGA database mRNAseq_325. **p < 0.01. (D) IHC analyze the expression of POFUT1 in glioma patients of different grades. (E) Statistical analysis of IHC. The vertical axis shows the H‐score and the horizontal axis represents groups. **p < 0.01. Representative immunohistochemical staining of POFUT1 in glioma tissues of different WHO grades. GBM was included as the WHO grade IV group.

To investigate the expression of POFUT1 in glioma, we first analyzed data from the TCGA database, comparing glioblastoma multiforme (GBM) and low‐grade glioma (LGG) tissues with normal tissues. As shown in Figure 1A, POFUT1 expression was significantly higher in glioma samples than in normal controls. Among glioma subtypes, GBM tissues showed a further increase in POFUT1 expression compared with LGG tissues. We further validated this finding using two independent CGGA datasets (mRNAseq_693 and mRNAseq_325), which revealed that POFUT1 expression increased progressively with higher glioma malignancy grade (Figure 1B and C). To confirm these bioinformatic results, we performed immunohistochemistry (IHC) on 115 glioma samples obtained from Shanxi Provincial People's Hospital. IHC analysis further confirmed the upregulation of POFUT1 in glioma tissues relative to adjacent normal tissues, supporting the findings obtained from the TCGA and CGGA datasets (Figure 1D and E). Overall, our data show that POFUT1 is highly expressed in human gliomas, with higher expression levels observed in tumors of more advanced grade.

### 3.2 Elevated POFUT1 Expression Predicts Poor Prognosis in Glioma Patients

**FIGURE 2:**
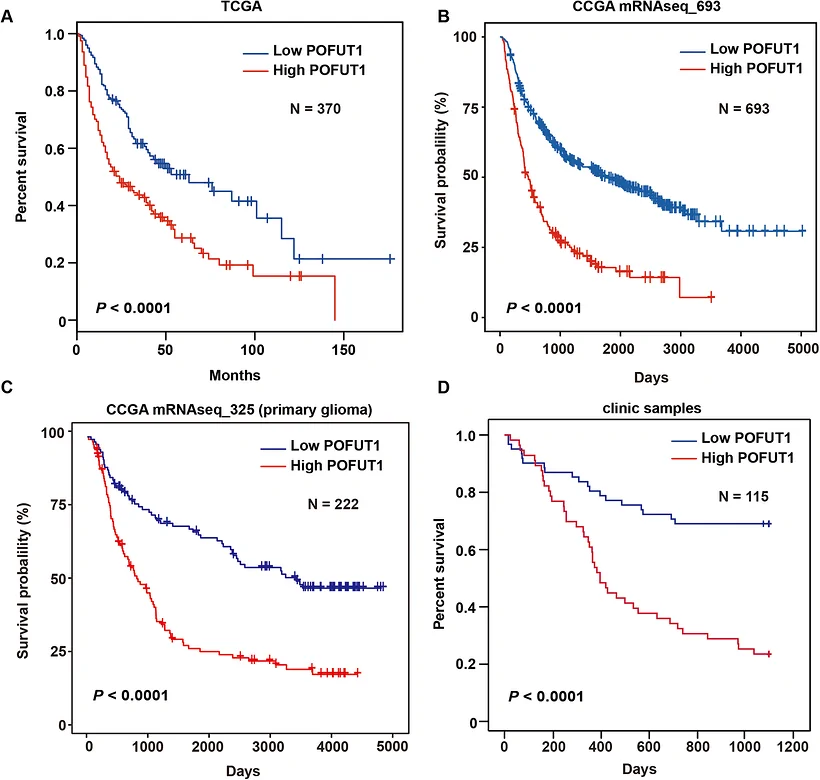
The survival analysis of POFUT1 in gliomas. (A) Kaplan–Meier survival analysis of POFUT1 in 370 GBM from TGCA database. (B) Kaplan–Meier survival analysis of POFUT1 from CGCA database mRNAseq_693. (C) Kaplan–Meier survival analysis of POFUT1 from CGCA database mRNAseq_325. (D) Kaplan–Meier survival analysis of POFUT1 in 115 glioma patients. The high expression of POFUT1 is highlighted in red, and the low expression of POFUT1 is highlighted in blue. The vertical axis shows the survival rate and the horizontal axis represents time.

We next examined whether POFUT1 expression was related to patient survival in the TCGA dataset. Patients with high POFUT1 expression exhibited significantly poorer overall survival than those with low POFUT1 expression (Figures 2A and S1). This finding was consistently validated in two independent CGGA cohorts (mRNAseq_693 and mRNAseq_325; Figure 2B and C). This prognostic association was further validated in our clinical cohort of 115 glioma cases, in which high POFUT1 expression was linked to reduced patient survival (Figure 2D). And, survival analysis was performed only in the 115 glioma patients with available clinicopathological and follow‐up information. Subgroup analysis indicated that high POFUT1 expression remained correlated with worse survival across diverse patient strata, including those categorized by age, gender, Ki‑67 index, MGMT promoter status, 1p/19q co‑deletion, and IDH1 mutation (Figure S2). Collectively, these results demonstrate that elevated POFUT1 expression is significantly associated with adverse prognosis in glioma.

### 3.3 POFUT1 Expression Independently Predicts Poor Prognosis in Glioma

**FIGURE 3:**
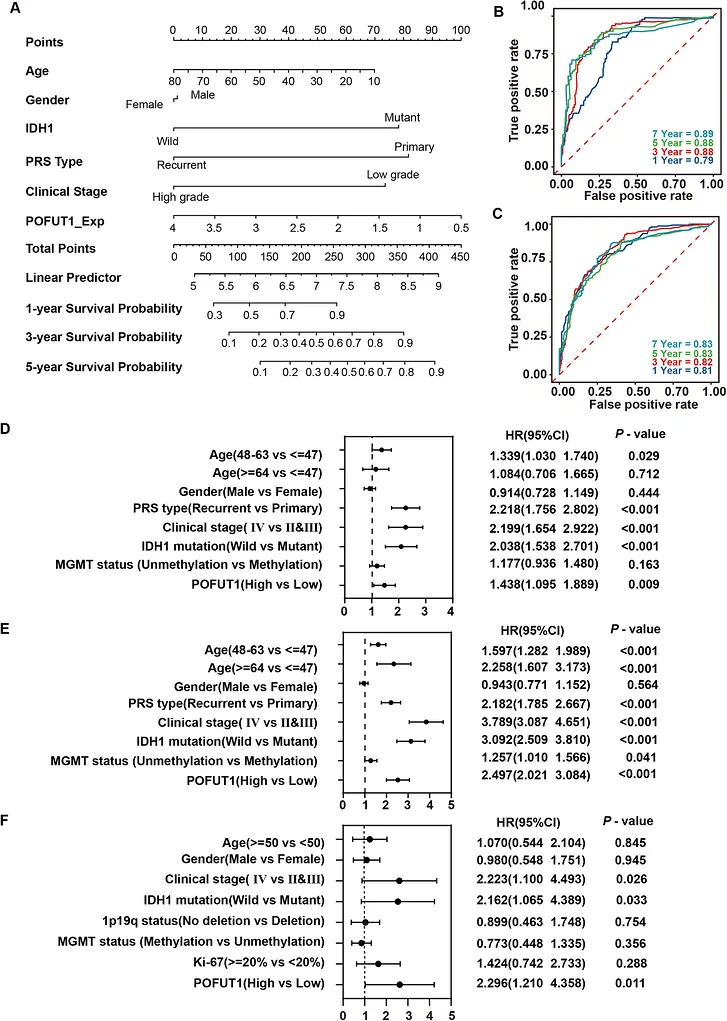
Construction and validation of a POFUT1‐integrated prognostic model for glioma patients. (A) Nomogram incorporating age, tumor type, clinical stage, IDH1 mutation status, MGMT promoter methylation status, and POFUT1 expression to predict 1‑, 3‑, and 5‑year overall survival probability. (B) Time‑dependent receiver operating characteristic (ROC) curves of the nomogram for predicting survival at 1, 3, and 5 years. (C) Comparison of ROC curves between the POFUT1‑integrated nomogram (red line) and a baseline model containing only conventional clinicopathological variables (blue line). (D) Multivariate Cox analysis in TCGA showing the independent prognostic value of POFUT1. (E) Multivariate Cox analysis in CGGA showing the independent prognostic value of POFUT1. (F) Multivariate Cox analysis in our clinical cohorts showing the independent prognostic value of POFUT1. HR, hazard ratio; CI, confidence interval.

To construct a prognostic model, a nomogram integrating age, tumor type, clinical stage, IDH1 mutation, MGMT promoter methylation, and POFUT1 expression was developed to predict 1‑, 3‑, and 5‑year overall survival (Figure 3A). The predictive ability of the model was further evaluated using time‐dependent receiver operating characteristic (ROC) curves. The area under the curves (AUC) for 1‐, 3‐, and 5‐year survival were 0.83, 0.85, and 0.84, respectively, indicating good prediction accuracy (Figure 3B). The model incorporating POFUT1 exhibited significantly better discriminative ability than a baseline model containing only conventional clinicopathological variables (Figure 3C). Multivariate Cox regression analysis using the TCGA dataset further identified high POFUT1 expression as an independent risk factor for poor survival (Figure 3D). Consistent results were obtained in the CGGA dataset, where high POFUT1 expression, along with IDH mutation status, clinical stage, PRS type, and age, was significantly associated with worse outcomes (Figure 3E). The independent prognostic value of POFUT1 was also validated in our cohort of 115 clinical glioma samples (Figure 3F). Based on the available clinicopathological variables and POFUT1 expression, we constructed a preliminary prognostic model for glioma patients.

### 3.4 POFUT1 Expression Is Associated With Clinicopathological Features in Glioma Patients

To further clarify the clinical implications of POFUT1, we assessed whether its expression was related to key clinicopathological parameters. Based on POFUT1 expression levels, patients were divided into high‐expression (POFUT1‐_high)_ and low‐expression (POFUT1‐_low_) groups using an ROC curve‐defined threshold. Notably, in the CGGA dataset, POFUT1 levels were significantly associated with grade, IDH status, patient survival, and primary/recurrent disease (Table 1). Strikingly, a consistent pattern was observed in our in‐house cohort of 115 patients, where POFUT1 expression correlated with age, grade, IDH status, 1p19q codeletion, Ki‐67 index, and survival outcome (Table 2). These data establish POFUT1 as a factor significantly linked to critical clinicopathological determinants in glioma.

**TABLE 1:** Correlation analysis between POFUT1 expression and clinical information in CGGA mRNAseq_693 dataset.

Clinical features		POFUT1_low_	POFUT1_high_	*p* Value
Gender	Male	296	102	0.3503
	Female	210	85	
Age	≤47	336	125	0.2617
	48‐63	140	45	
	≥64	30	17	
WHO Grade	II	166	23	**0.0000**
	III	199	56	
	IV	142	108	
IDH type	Wild type	282	75	**0.0000**
	Mutant type	175	111	
MGMT type	methylation	243	72	0.3388
	Unmethylated	167	60	
Survival status	Alive	230	36	**0.0000**
	Dead	257	140	
Onset state	Primary	328	95	**0.0007**
	Recurrent	179	93	

The bold values in indicate *P* values < 0.05, representing statistically significant differences.

**TABLE 2:** Correlation analysis between POFUT1 and clinicopathological information in clinical samples.

Clinical features		POFUT1_low_	POFUT1_high_	*p* Value
Gender	Male	36	26	0.1730
	Female	24	29	
Age	<50	40	15	**0.0000**
	≥50	20	40	
WHO Grade	II	28	0	**0.0000**
	III	27	10	
	IV	5	45	
IDH type	Wild type	30	45	**0.0000**
	Mutant type	30	10	
1p19q type	Deletion	31	10	**0.0000**
	No deletion	29	45	
MGMT type	methylation	36	24	0.0810
	Unmethylated	24	31	
Ki67	<20%	35	7	**0.0000**
	≥20%	25	48	
Survival status	Alive	41	12	**0.0000**
	Dead	20	43	

The bold values in indicate *P* values < 0.05, representing statistically significant differences.

### 3.5 POFUT1 Promotes Malignant Phenotypes of Glioma In Vivo and In Vitro

**FIGURE 4:**
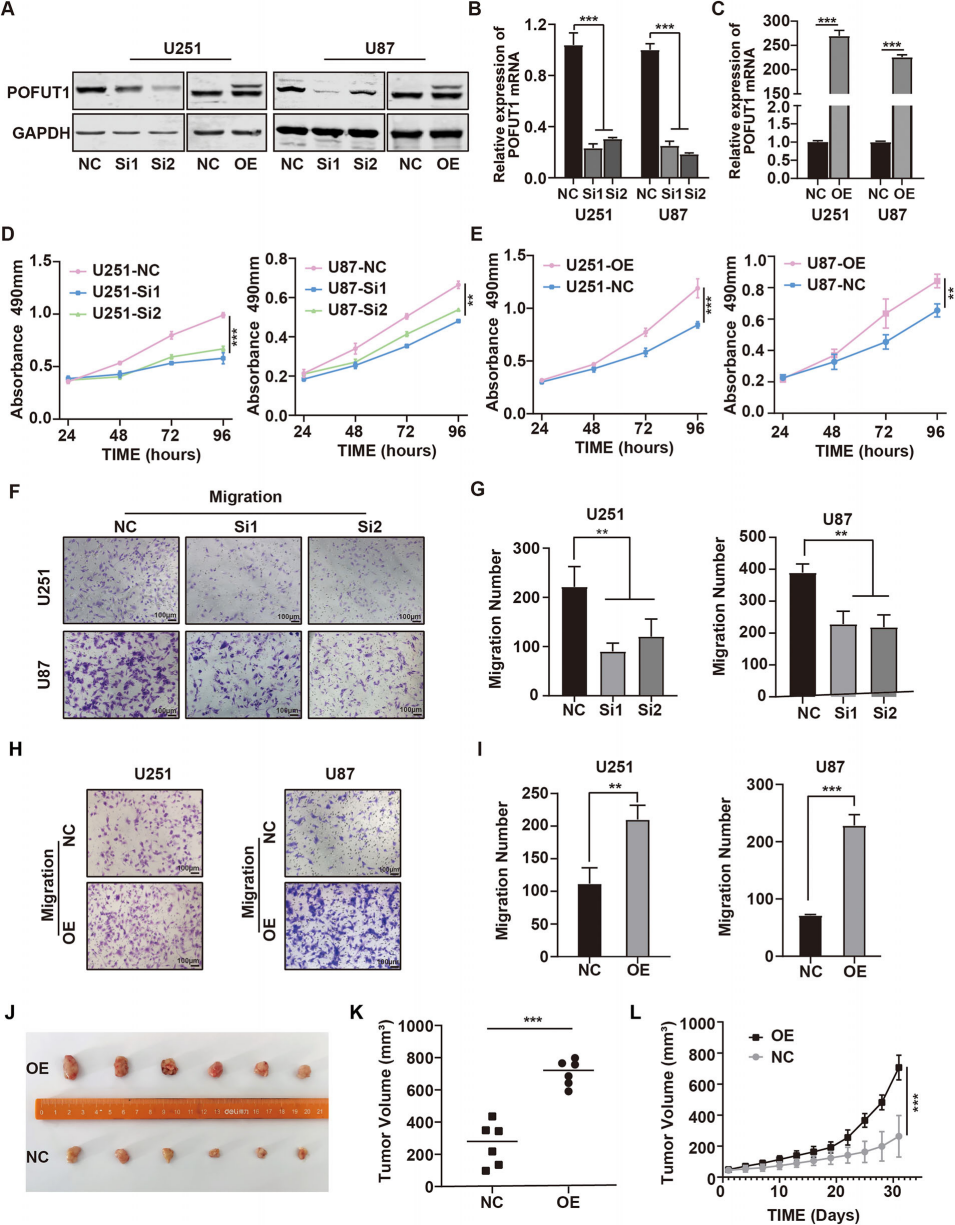
The effect of POFUT1 on proliferation, migration and invasion in GBM cells. (A) Western blot detected the efficiency of knockdowned and overexpressed POFUT1 in U251 and U87 cells. (B) RT‐qPCR analyzed the efficiency of knockdowned POFUT1 in U251 and U87 cells. ***p < 0.001. (C) RT‐qPCR analyzed the efficiency of overexpressed POFUT1 in U251 and U87 cells. ***p < 0.001. (D) MTT assay detected the proliferative ability of knockdown POFUT1 in U251 and U87 cells. **p < 0.01, ***p < 0.001. (E) MTT assay detected the proliferative ability of knockdown POFUT1 in. ***p < 0.001. (E) MTT assay detected the proliferative ability of overexpress POFUT1 in U251 and U87 cells. **p < 0.01, ***p < 0.001. (F) Transwell assay detected the migratory ability of knockdown POFUT1 in U251 and U87 cells. Bar = 100 µm. G. Statistical analysis of migratory ability. **p < 0.01. (H) Transwell assay detected the migratory ability of overexpress POFUT1 in U251 and U87 cells. Bar = 100 µm. (I) Statistical analysis of migratory ability. **p < 0.01, ***p < 0.001. (J) The picture of original tumor tissues about mouse xenograft model. (K) The tumor volume of mouse xenograft model. ***p < 0.001. (L) The growth curve of the xenograft model. ***p < 0.001.

To investigate the functional role of POFUT1 in glioma, we performed knockdown and overexpression of POFUT1 in U251 and U87 cell lines. Western blot and RT‑qPCR analyses confirmed efficient knockdown and successful overexpression of POFUT1 (Figure 4A–C). Functional assays further demonstrated that POFUT1 depletion suppressed the growth of U251 and U87 cells, as indicated by the MTT results (Figure 4D). In contrast, forced expression of POFUT1 increased the proliferative capacity of glioma cells (Figure 4E). Similarly, Transwell migration assays demonstrated reduced migratory ability following POFUT1 knockdown (Figure 4F and G) and increased migration upon POFUT1 overexpression in U251 and U87 cells (Figure 4H and I). To further confirm the biological role of POFUT1 in vivo, we generated a subcutaneous xenograft model with U251 cells stably expressing POFUT1 (OE) or the corresponding control vector (NC). Tumors derived from OE cells exhibited larger sizes (Figure 4J and K) and increased volumes (Figure 4L) compared with the NC group. Overall, our data demonstrate that POFUT1 contributes to glioma progression by promoting cell proliferation, migration, and tumor growth.

### 3.6 POFUT1 Activates the PI3K‐AKT Pathway in Glioma

**FIGURE 5:**
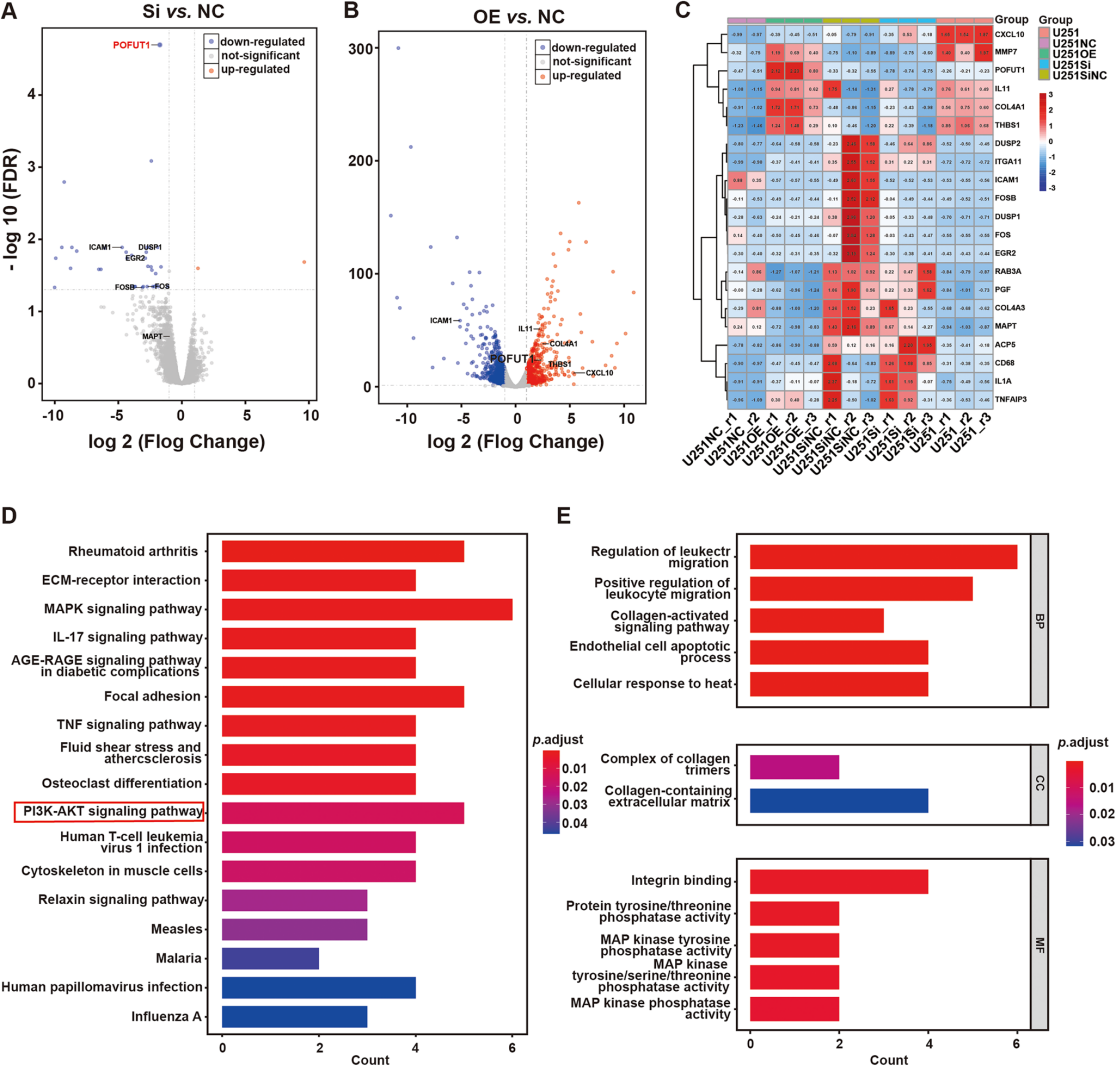
The expression of POFUT1 is associated with PI3K‐AKT pathway. (A) Volcano map showed the different genes between the NC and OE groups in U251. (B) Volcano map showed the different genes between the NC and Si groups in U251. (C) Heatmap showed the expression of different genes in each group. (D) KEGG enrichment analysis showed the different pathways. (E) KEGG enrichment analysis showed the different pathways.

To further define the mechanisms underlying the pro‐tumor effects of POFUT1, transcriptome profiling was conducted in U251 cells following POFUT1 silencing and overexpression. Volcano plots and heatmaps revealed substantial changes in gene expression upon POFUT1 manipulation (Figure 5A–C). Further KEGG and GO analyses indicated that the PI3K‐AKT signaling pathway was significantly enriched among the identified differentially expressed genes (Figure 5D and E).

### 3.7 POFUT1 Regulates Glioma Progression via PI3K‐AKT Signaling Pathway

**FIGURE 6:**
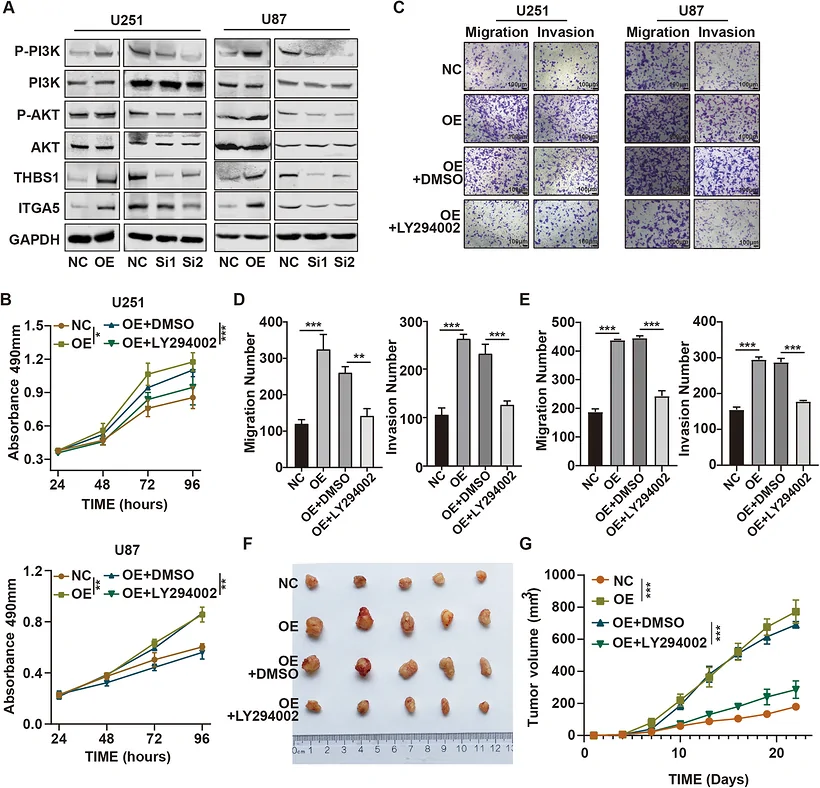
The malignant progression influenced by POFUT1 via PI3K‐AKT signaling pathway. (A) Western blot showed the change of biomarkers about PI3K‐AKT pathway in U251 and U87 cells. (B) MTT assay detected the proliferative ability of LY29400 after overexpressed POFUT1 in U251 and U87 cells. *p < 0.05, **p < 0.01, ***p < 0.001. (C) Transwell assay detected the migratory and invasive abilities of LY29400 after overexpressed POFUT1 in U251 and U87 cells. (D, E) Statistical analysis of migratory and invasive abilities in U251 and U87 cells. **p < 0.01, ***p < 0.001. (F) Representative images of excised xenograft tumors derived from U251 cells in the indicated groups. (G) Tumor growth curves in the U251 xenograft model. ***p < 0.001.

To further clarify the involvement of PI3K‐AKT signaling in POFUT1‐driven glioma progression, key proteins in this pathway were examined in U251 and U87 cells following POFUT1 knockdown and overexpression. Western blot analysis showed that POFUT1 knockdown reduced the levels of p‑PI3K, p‑AKT, THBS1, and ITGA5, while total PI3K and AKT remained unchanged (Figure 6A). Conversely, POFUT1 overexpression increased the phosphorylation of PI3K and AKT, as well as the expression of THBS1 and ITGA5 (Figure 6A). To further confirm the functional dependence on this pathway, we treated POFUT1‑overexpressing U251 and U87 cells with LY294002, a specific PI3K inhibitor. The inhibitor effectively reversed the pro‑proliferative, pro‑migratory, and pro‑invasive effects induced by POFUT1 overexpression (Figure 6 B–E). To further validate these findings in vivo, a xenograft model was established using U251 cells. In this model, treatment with a PI3K inhibitor significantly attenuated the tumor‐promoting effect induced by POFUT1 overexpression (Figure 6F and G). Taken together, these data support a tumor‐promoting role of POFUT1 in glioma and suggest that this effect is closely associated with activation of PI3K‐AKT signaling.

## 4 Discussion

**FIGURE 7:**
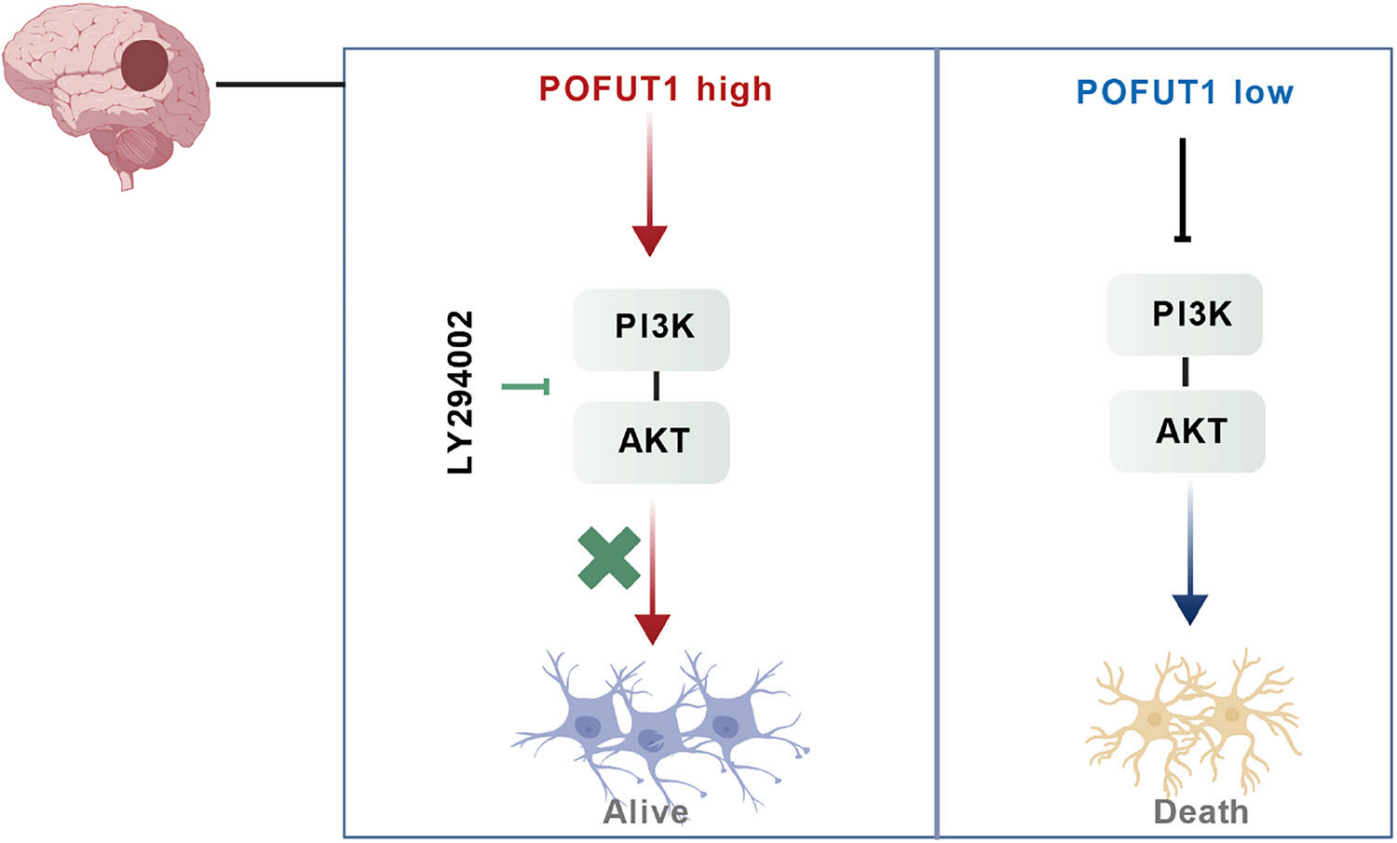
POFUT1 affects glioma progression via the PI3K‐AKT pathway.

In summary, our findings reveal an important role for POFUT1 in glioma progression. POFUT1 was markedly increased in glioma tissues and showed close associations with malignant clinicopathological characteristics, shorter survival, and poor patient prognosis. Functional experiments further demonstrated that POFUT1 enhanced glioma cell progression. Mechanistically, transcriptome sequencing together with subsequent validation indicated that POFUT1 may promote these oncogenic phenotypes through activation of the PI3K‐AKT signaling pathway (Figure 7). These results suggest that POFUT1 may serve as a promising prognostic biomarker and a potential therapeutic target for glioma.

The prognostic value of POFUT1 has been reported in several malignancies. Previous studies have suggested that POFUT1 may function as a prognostic predictor and therapeutic target in low‐grade gliomas (Yu et al. 2024). POFUT1‐related plasma biomarkers have also shown diagnostic potential in lung cancer (Leng et al. 2018), and POFUT1 has been proposed as a candidate biomarker in gastric cancer (Dong et al. 2017). These observations are supported by our clinical analyses, which demonstrated that high POFUT1 expression was closely associated with poorer survival outcomes in glioma patients. Moreover, the prognostic model incorporating POFUT1 expression showed favorable predictive performance, suggesting that POFUT1 may provide additional prognostic information for glioma risk stratification. However, several established molecular alterations, including chromosome 10q loss or monosomy 10, EGFR copy number gain, CDKN2A/2B deletion, TP53 mutation, and PTEN mutation, were not incorporated into the current model because complete molecular data were not available for all patients (Stichel et al. 2018). Therefore, larger cohorts with more comprehensive molecular profiling are needed to further refine and validate the prognostic utility of POFUT1.

Beyond its prognostic relevance, POFUT1 may actively contribute to malignant tumor phenotypes. In gastric cancer, POFUT1 has been shown to promote tumor progression through activation of Notch and Wnt signaling pathways (Dong et al. 2023). Increased POFUT1 expression also enhances peritumoral nerve invasion in head and neck squamous cell carcinoma (Barlak et al. 2023), while its upregulation in gastric cancer tissues has been linked to invasive behavior (Dong et al. 2017). These observations are consistent with our experimental findings. In glioma cells, POFUT1 overexpression promoted proliferation, migration, and invasion, whereas POFUT1 knockdown produced the opposite effects. The in vivo xenograft experiments further confirmed that POFUT1 facilitated tumor growth. Together, these results indicate that POFUT1 is not merely a prognostic marker but may also functionally participate in glioma progression.

Tumor progression is rarely driven by a single molecular alteration; instead, it usually reflects the coordinated activation of multiple signaling networks. In our study, the PI3K‐AKT pathway emerged as a major pathway associated with POFUT1‐mediated glioma malignancy. As one of the signaling pathways most frequently disrupted in tumors, PI3K‐AKT signaling contributes to multiple malignant behaviors, including uncontrolled growth, enhanced survival, migration, invasion, metabolic adaptation, drug resistance, and metastasis (Mohamed et al. 2022). Similarly, prior evidence indicates that POFUT1 can drive trophoblast cell progression, at least partly through MAPK and PI3K‐AKT pathway activation (Liu et al. 2017), and it has also been linked to proliferation and metastasis in esophageal cancer cells (Xu et al. 2021). In agreement with previous evidence, we found that forced POFUT1 expression increased the activation of PI3K‐AKT signaling in glioma cells. More importantly, blocking PI3K activity with LY294002 partially reversed the growth‐promoting effect of POFUT1 overexpression in vivo, suggesting that PI3K‐AKT signaling contributes to the oncogenic role of POFUT1. These data suggest that PI3K‐AKT activation is, at least in part, responsible for the oncogenic effect of POFUT1 in glioma.

Although our findings reveal a close association between POFUT1 and PI3K‐AKT activation, the molecular link between them remains to be fully defined. POFUT1 is responsible for adding O‐linked fucose to epidermal growth factor‐like repeat domains in multiple target proteins, including members of the Notch receptor family (He et al. 2024). By regulating receptor maturation, ligand recognition, and downstream signal transduction, POFUT1 may influence Notch‐dependent biological processes. Given that Notch signaling has been implicated in glioma cell proliferation, invasion, stemness maintenance, and therapeutic resistance, it is plausible that POFUT1 contributes to glioma progression through a Notch‐related mechanism. In addition, our analysis identified THBS1 and ITGA5 as potential downstream responsive proteins associated with POFUT1 expression. Notably, both proteins participate in extracellular matrix remodeling and cell adhesion‐related processes, including integrin‐mediated signaling, which may ultimately promote PI3K‐AKT activation (Lu et al. 2024). Based on these observations, POFUT1 may enhance PI3K‐AKT signaling through a Notch‐dependent route and/or through a THBS1/ITGA5‐associated extracellular matrix‐integrin axis. However, this interpretation remains hypothetical. Further rescue experiments targeting THBS1, ITGA5, and Notch‐related components are required to determine whether these molecules directly mediate the effect of POFUT1 on PI3K‐AKT activation and glioma malignancy. The mechanisms underlying POFUT1 upregulation in glioma also deserve further investigation. POFUT1 expression may be shaped by transcriptional regulation, epigenetic alterations, non‐coding RNAs, or signals derived from the tumor microenvironment (Ullah et al. 2026). Clarifying these upstream regulatory events would provide a more complete view of how POFUT1 becomes dysregulated during glioma progression. Such studies may also uncover additional therapeutic opportunities within the broader POFUT1‐centered regulatory network.

Despite these findings, several limitations need to be acknowledged. First, the clinical samples used for immunohistochemical and prognostic analyses were obtained from a single center, which may introduce selection bias. Second, analyses based on public databases were mainly cross‐sectional and therefore could not establish a causal relationship between POFUT1 expression and glioma progression. Finally, although our functional experiments and LY294002 rescue assays support the involvement of PI3K‐AKT signaling, the detailed regulatory relationships among POFUT1, Notch signaling, THBS1/ITGA5, and the PI3K‐AKT pathway remain to be experimentally verified.

## 5 Conclusion

Overall, our findings suggest that POFUT1 plays a tumor‐promoting role in glioma. Its elevated expression was associated with worse clinical prognosis, while experimental evidence demonstrated that POFUT1 facilitated glioma cell growth, motility, invasiveness, and xenograft tumor formation. Further mechanistic analysis indicated that activation of the PI3K‐AKT pathway may be involved in POFUT1‐mediated malignant phenotypes. Therefore, POFUT1 may serve as a promising prognostic indicator and a potential molecular target for glioma treatment.

## Author Contributions

**Xiaochen Yang**: investigation. **Aijie Guo**: formal analysis. **Yang Mi**: investigation. **Yifan Wang**: methodology, software. **Chunlei Ju**: validation. **Chunhong Wang**: supervision, writing – review and editing, project administration. **Hongming Ji**: funding acquisition, supervision, writing – review and editing, project administration, resources. **Kai Yang**: writing – original draft, data curation, conceptualization, methodology, visualization. **Yifei Su**: investigation.

## Funding

This work was supported by Fundamental Research Program of Shanxi Provinc (202203021222362) (202403021211136), the Central Government Guiding Fund for Local Science and Technology Development Projects (YDZJSX2022B013), and Shanxi Provincial Collaborative Project on Integrated Traditional Chinese and Western Medicine for Major and Complex Diseases.

## Ethics Statement

Human tissue collection and all animal experiments were reviewed and approved by the Ethics Committee of Shanxi Provincial People's Hospital, Shanxi Medical University, China (Approval No. 2021 Provincial Medical Ethics Review No. 320). All procedures involving human samples and animals were conducted in accordance with the approved institutional guidelines and relevant ethical regulations. Written informed consent was obtained from all patients prior to the collection of clinical tissue samples.

## Conflicts of Interest

The authors declare no competing interests.

## Supporting information




**Table S1**. Clinical information of Glioma patients in the study.

**Table S2**. siRNA sequences and primers of POFUT1.

**Figure S1**. Stratified survival analysis in TCGA dataset. (A) Stratified analysis was performed based on IDH1 mutation status (mutant vs. wild‐type). (B) Stratified analysis was performed based on LGG and GBM. (C) Stratified analysis was performed based on age. (D) Stratified analysis was performed based on primary tumor and recurrent tumor. (E) Stratified analysis was performed based on sex. (F) Stratified analysis was performed based on MGMT.

**Figure S2**. Stratified survival analysis in 115 Glioma samples. (A) Stratified analysis was performed based on age. (B) Stratified analysis was performed based on sex. (C) Stratified analysis was performed based on Ki67 expression. (D) Stratified analysis was performed based on MGMT. (E) Stratified analysis was performed based on 1q19p deletion. (F) Stratified analysis was performed based on IDH1 mutation status (mutant vs. wild‐type).

## Data Availability

The transcriptomic sequencing data obtained from this study have been archived in the GEO repository and can be accessed using the accession ID GSE316535.
